# Anesthetic considerations in hyperparathyroid crisis: A case report

**DOI:** 10.1097/MD.0000000000024216

**Published:** 2021-01-08

**Authors:** Hao Kong, Zhen Zhang, Hong Zhang

**Affiliations:** Department of Anesthesiology, Peking University First Hospital, Beijing, China.

**Keywords:** anesthetic consideration, hypercalcemia, hyperparathyroid crisis, parathyroidectomy

## Abstract

**Introduction:**

Hyperparathyroid crisis is a rare and potentially life-threatening complication of severe calcium intoxication. Parathyroidectomy is the only curative method for hyperparathyroid crisis. Several case reports and case series have been published on the medical and surgical treatments for hyperparathyroid crisis, however, few reports have focused on the associated perioperative anesthetic management.

**Patient concerns:**

A 48-year-old Chinese woman presented with a 2-week history of nausea and vomiting and complained of mental status alteration including confusion and agitation in the 24 hours prior to her admission. She denied any history of past illness. Laboratory tests showed severe hypercalcemia crisis with a serum calcium level of 5.21 mmol/L and a serum intact parathyroid hormone level of > 5000 pg/mL.

**Diagnosis:**

The diagnosis was hyperparathyroid crisis, acute kidney injury, acute liver injury, rhabdomyolysis, infection, and shock.

**Interventions:**

She underwent initial management with aggressive intravenous fluid resuscitation, loop diuretic treatment, vitamin D supplement, intravenous bisphosphonates, and calcitonin therapy. However, her condition worsened, and she was transferred to the operating theater for a parathyroidectomy under general anesthesia. She was under general anesthesia and monitored with electrocardiogram, pulse oxygen saturation, continuous arterial blood pressure, central venous pressure and nasopharyngeal temperature. Cardiac output and stroke volume variation were monitored from the FloTrac system. After liberal fluid rehydration, circulatory support, cooling treatment and calcium supplement after tumor removal, her unstable vital signs gradually improved.

**Outcomes:**

After meticulous anesthetic management by the anesthesiologist and complete tumor resection by the surgeon, she survived this fatal disease. The patients was discharged on postoperative day 37 without any sequelae.

**Lessons:**

Patients with hyperparathyroid crisis should undergo a thorough preoperative evaluation. Difficult airway, fluid depletion, multiple organ dysfunction, hypercoagulability, and concomitant diseases are the primary challenges in anesthetic management. After tumor removal, the serum calcium level should be monitored closely and calcium should be supplemented in a timely manner to prevent serious complications.

## 1 Introduction

Hyperparathyroid crisis is a rare but life-threatening endocrine emergency with an incidence ranging from 2.7% to 6.7% in patients having hyperparathyroidism.^[1–4]^ Although the diagnostic criteria for hypercalcemic crisis are not yet well established, the most generally accepted criteria include an elevated serum intact parathyroid hormone level (iPTH) together with a substantial increase in the serum calcium level higher than 3.5 mmol/L, associated with an acute onset of symptoms.^[5]^

Presentations of hyperparathyroid crisis are heterogeneous, including nausea, vomiting, fatigue, altered sensorium, dehydration, decreased renal function, cardiac arrhythmias, mental alteration, confusion, and coma; if the condition is untreated, it may result in death.^[6,7]^ The treatment of hyperparathyroid crisis begins with aggressive hydration, diuresis, and calcitonin and bisphosphonate administration; dialysis is used as salvage therapy when the above options failed. Parathyroidectomy is the only curative method for hyperparathyroid crisis. It involves the removal of the overactive gland, thereby lowering the levels of iPTH released into the blood and decreasing serum calcium levels.^[5]^ The mortality rate associated with hyperparathyroid crisis has gradually declined in recent years due to early diagnoses and improved medical interventions. However, fluid depletion, electrolyte disorders, and multiple organ damage remain great challenges for anesthetic management of patients with hyperparathyroid crisis.

Here, we present a rare case in which a patient with hyperparathyroid crisis survived and aim to provide some anesthetic considerations from the perspectives of anesthesiologists.

## 2 Case presentation

A 48-year-old Chinese woman presented with a 2-week history of nausea and vomiting and complained of mental status alteration including confusion and agitation in the 24 h prior to her admission. She denied any history of past illness. She was tachycardic (140 bpm) and hypertensive (160/100 mm Hg) with an increased respiratory rate (31/min) and mild hyperthermia (37.3°C). Laboratory tests showed severe hypercalcemia crisis with a serum calcium level of 5.21 mmol/L (normal range [NR]: 2.11–2.52 mmol/L) and a serum iPTH level of > 5000 pg/ml (NR: 15–65 pg/ml). Laboratory investigations (Table 1) revealed multiple organ damage, supporting the diagnosis of acute kidney injury, liver injury, and myocardial injury. Rhabdomyolysis was suggested by markedly increased myokinase and myoglobin levels. Procalcitonin level and leukocyte count were significantly increased, indicating an infection. The patient also presented with hypokalemia, hypophosphatemia, alkalosis, and hyperlactic acidemia. An electrocardiogram (ECG) showed atrial flutter with a high ventricular rate and a chest radiograph was unremarkable. An echocardiogram showed good function of myocardial contractility with a normal ejection fraction. Thyroid ultrasonography revealed a separated lesion of 5.4 × 3.0 × 3.0 cm^3^ in size with plentiful blood flow in the inferior pole of the left lobe.

**Table 1:** Results of the initial laboratory investigations.

Investigation	Test	Result	Normal range
Biochemical tests	Serum calcium	5.21	2.11–2.52 mmol/L
	Intact parathyroid hormone level	> 5000	15–65 pg/ml
	25-OH-VitD	13.56	75–250 nmol/L
	Blood urea nitrogen	16.20	1.8–7.1 mmol/L
	Creatinine	191.60	44–133 μmol/L
	Alanine aminotransferase	234	7–40 IU/L
	Aspartate aminotransferase	541	13–35 IU/L
	γ-glutamyl transpeptadase	141	7–45 IU/L
	Total bilirubin	80.4	1.7–20 μmol/L
	Hypersensitive troponin I	7.541	0–0.03 ng/ml
	Creatine kinase-myoglobin	27.7	< 5 ng/ml
	Creatine kinase	> 8000	25–170 IU/L
	Myoglobin	> 500	0–100 ng/ml
	Serum potassium	1.7	3.5–5.5 mmol/L
	Serum phosphorus	0.66	0.85–1.51 mmol/L
	Procalcitonin	13.69	< 0.5 ng/ml
Full blood count	White blood cell count	30.9 × 110^9^	3.5–9.5 × 10^9^/L
	Hemoglobin	128	115–150 g/L
Blood gas analysis	PH	7.53	7.35–7.45
	PO_2_	86	75–100 mm Hg
	PCO_2_	33	35–45 mm Hg
	HCO_3_^-^	27.6	22–27 mmol/L
	Lactic acid	7.6	0.5–1.7mmol/L

She underwent initial management with aggressive intravenous fluid resuscitation, loop diuretic treatment, vitamin D supplement, intravenous bisphosphonates, and calcitonin therapy. Empiric broad-spectrum antibiotic therapy was initiated despite the origin of the infection being unclear. However, a poor response was achieved. The serum calcium level remained higher than 5 mmol/L after 12 h of treatment and the patient was in a coma. Therefore, continuous renal replacement therapy with free calcium dialysate was considered. After 48 h of continuous renal replacement therapy, the serum calcium level gradually decreased to 3.53 mmol/L. However, the patient's symptoms and vital signs deteriorated. During this period, her pulse oxygen saturation decreased and non-invasive mechanical ventilation had to be initiated. A chest X-ray showed multiple bilateral infiltrates. Furthermore, her blood pressure dropped to 75/40 mmHg and her heart rate gradually increased to 170 bpm; therefore, continuous norepinephrine infusion (12 μg/min) was started. Given the confusing and disappointing outcome, urgent parathyroidectomy was performed based on a multidisciplinary consultation. The patient was intubated in the emergency intensive care unit after infusion of 2 mg midazolam and 50 mg rocuronium, and then transferred to the operating theater.

In the operating room, 2 venous accesses were established using a peripheral venous catheter and a central venous hemodialysis catheter. The left radial artery was cannulated for real-time arterial blood pressure monitoring and intermittent blood sampling. Meanwhile, the FloTrac system was used to monitor the cardiac output and stroke volume variation. Central venous pressure was measured via 1 lumen of the central venous hemodialysis catheter. The bispectral index was monitored with a value of 30 and nasopharyngeal temperature indicated of 40.0°C. Six ice packs were placed in both of the patient's armpits, both sides of the groin, and popliteal fossae. Norepinephrine was pumped continuously (30 μg/min); the patient was struggling, with her BP and HR being 100/50 mmHg and 160 beats/min, respectively. Quick and cursory echocardiography of the inferior vena cava (IVC) diameter was performed at end-expiration and end-inspiration. The distensibility index of the IVC was calculated as > 20%. Considering the patient's daily positive fluid balance was approximately 1000 ml, as informed by her physician, combined with unstable hemodynamics, infection, high body temperature, and a stroke volume variation of 19%, we believed her volume depletion had not been corrected. Therefore, an aggressive fluid replacement was considered, to which the hemodynamic response was favorable. Anesthesia was maintained with intravenous remifentanil (target-controlled infusion with an effect-site level of 2 ng/ml) and sevoflurane (end-tidal concentration: 0.8%) with an inspired oxygen concentration of 75%. Sufentanil (total 5 μg) was infused at the start of the surgery, and rocuronium (total 40 mg) was intermittently injected. The calcium level was closely monitored by blood gas analysis. At the beginning of the surgery, the ionized calcium level was 2.20 mmol/L (NR 1.19–1.35 mmol/L). After tumor removal, the ionized calcium level dropped to 1.90 mmol/L and 1 g of calcium chloride was added to the fluids to prevent a rapid decline of the calcium level. The operation lasted 87 min and the tumor was completely removed. At the end of the surgery, the patient's BP was 120/80 mmHg and HR was 135 bpm (with 20 μg/min norepinephrine). Her body temperature dropped to 38.0°C and ionized calcium level was 1.73 mmol/L. She received 4 L of crystalloids, 1 L of succinyl gelatin, and 0.1 L of albumin. Blood loss and urine output were 20 ml and 800 ml, respectively. She was transferred to the intensive care unit.

After surgery, she was monitored closely in case hypocalcemia. High doses of calcium supplements and vitamin D were administered intravenously. Phosphorus, magnesium, and potassium were supplemented to prevent electrolyte disturbances. Chest X-ray revealed a large infiltrate in the left lung lobe, the most likely cause of which, as determined by physicians, aspiration pneumonia; therefore, antibiotic therapy was upgraded. Despite tachycardia and elevated myocardial enzyme and troponin I levels, echocardiography showed good systolic function with an ejection fraction of 70%. The patient regained consciousness and her body temperature normalized, however, new gastrointestinal bleeding was found on postoperative day (POD) 3. Three days after proton pump inhibitors administration and transfusion therapy, the patient's fecal occult blood turned negative. Continuous norepinephrine infusion was withdrawn on POD 5. The patient did not show oliguria or anuria, and her renal and liver functions gradually recovered. On POD 6, the patient was extubated. On POD 13, the patient was transferred to the ward and discharged from the hospital on POD 37. Histological examination revealed an encapsulated parathyroid adenoma with no local, capsular, or vascular invasion and no other feature suggestive of malignancy.

## 3 Discussion

We report a rare case of a patient with hyperparathyroid crisis characterized by an extremely high serum iPTH level who survived. In the anesthetic management of hyperparathyroid crisis, some points should be taken into consideration.

The optimal timing of the surgery in this scenario has not been definitively established because of the difficulty of comparing these rare cases. Based on the limited studies available, an emergent surgery, that is, prompt parathyroidectomy after diagnosis, is not recommended, owing to unstable vital signs caused primarily by hypercalcemia and dehydration.^[2,5,8,9]^ Rehydration and calcium-lowering treatments should be used initially and simultaneously to provide an effective bridge to parathyroidectomy.^[2]^ Another controversy stems from an early (<48–72 h) or a delayed (>72 h) surgery. In a previous study, Lew et al. included data for 35 years and reported no significant differences in long-term survival for patients who underwent surgery within 72 hours or not.^[8]^ However, for patients with a poor medical response, sufficient hydration and early parathyroidectomy within 48 h are highly advised.^[9]^ Therefore, early surgical intervention might be better for patients who are resistant to medicine, but not mandatory for patients with an effective medical response. Our patient underwent surgery in the 68th hour after admission. Considering the occurrence of aspiration pneumonia and frustrating response of calcium-lowering treatments, an earlier tracheal intubation and surgical intervention might be beneficial for her.

A thorough and meticulous assessment of volume status is pivotal for intraoperative fluid therapy and hemodynamic management. In patients admitted with severe dehydration; presenting with vomiting; or being treated with aggressive fluids, diuretics, and/or dialysis, ensuring an accurate calculation of the daily liquid balance is not easy. In addition, insensible fluid loss should be considered, especially when it is accompanied by hyperthermia (increased skin evaporation) or infection (water entering the third space through increased vascular permeability). Ultrasonographic assessment of the diameter of the IVC^[10]^ combined with the size of the heart cavity and papillary muscle kissing sign is a rapid and non-invasive method to assess volume status. Volume depletion is best corrected before induction; otherwise, hypotension or circulatory failure after anesthetic induction may develop at a remarkably higher frequency. Our patient had an insufficient volume before surgery; therefore, extensive fluid infusion was performed during the surgery.

There is no recognized calcium level considered to be safe before surgery; a level of < 3 mmol/L was recommended by Singh et al.^[11]^ The serum calcium level should be closely monitored during the operation. After tumor removal, the serum calcium level may decrease rapidly within a few minutes. Myocardial infarction, visual and auditory hallucinations, and creeping numbness were reported to be associated with a rapid reduction of serum calcium level, even in patients with normal serum calcium levels.^[12]^ In a previous study conducted by Harjit et al,^[12]^ after successful tumor removal, vigorous intraoperative intravenous infusion of calcium was recommended for maintaining serum calcium levels just 1 mmol/L lower than the preoperative levels. The authors concluded a gradual and well-controlled decline of serum calcium was the cornerstone of surgical treatment for hyperparathyroid crisis to avoid fatal complications. In addition, we should not neglect chlorine, potassium, and phosphorus disturbances, which are often accompanied by abnormal serum calcium level, throughout the entire perioperative period.^[13]^

Hypercalcemia induces functional disturbances in a group of organs and the damage is not specific, but rather similar to that observed in many other diseases. The neurologic system, cardiovascular system, and kidney, with their susceptibility and vulnerability in hyperparathyroid crisis, were the focus of our preoperative evaluation. Central nervous system manifestations include confusion, poor concentration, and personality changes that range from irritability to lethargy and coma. Cardiac arrhythmias are common in patients with hyperparathyroid crisis. Hypercalcemia most often shortens ST-segment and consequently reduces QT interval.^[14]^ The presentations of severe cardiac arrhythmia can be highly variable, including tachy-brady syndrome, ventricular tachycardia, ventricular fibrillation, and paroxysmal atrioventricular block.^[15]^ Our patient showed a shortened QT interval before surgery, which resolved after parathyroidectomy. In addition, our patient's first ECG showed atrial flutter, which may be primarily related to hypokalemia and reverted after potassium supplementation. Persistent hypercalcemia can also cause myocardial injury and heart failure.^[16]^ Preoperative ECG and echocardiography are required to detect the function of cardiac conductivity and contractility. After tumor removal, the anesthesiologist should carefully monitor the circulation. Rapid serum calcium reduction can result in hypotension (loss of vascular tone), heart failure (impaired cardiac contractility), or myocardial infarction.^[12,14]^ Premature ventricular contractions and ventricular fibrillation can occur during severe hypocalcemia. Acute kidney injury is a frequent complication of hyperparathyroid crisis, and hypercalcemia impairs renal function in several different ways.^[14]^ Early polyuria may develop into oliguria and anuria. Most patients’ renal function will gradually recover after surgery. Acute liver injury is not common in hyperparathyroid crisis and the mechanism of injury is not completely clear. It might be associated with hepatic ischemia and hypoxia caused by hypoperfusion. Yu et al reported a case in which a patient with hepatitis C died of hepatic failure after parathyroidectomy for hyperparathyroid crisis.^[9]^ In addition, rhabdomyolysis secondary to hyperparathyroid crisis developed in this patient, was not reported previously. The pathogenesis is probably multifactorial. For example, metabolic and endocrinologic diseases such as hypokalemia and hypophosphatemia, as observed in the case of our patient, can cause rhabdomyolysis.^[17]^ It was previously reported that hyperthyroidism may cause rhabdomyolysis by increasing energy consumption, which is associated with the depletion of muscle energy stores and substrates.^[18]^ Rhabdomyolysis aggravates liver and kidney injury.

Patients with hyperparathyroid crisis were reported to have significantly larger glands, and difficult airway caused by compression and deviation of the tumor was reported in a patient with hyperparathyroid crisis in a previous study.^[19]^ Therefore, careful airway evaluation is necessary.

Regarding intraoperative monitoring, apart from standard monitoring measures for general anesthesia including ECG, non-invasive blood pressure measurement, pulse oximetry, and temperature measurement, real-time arterial blood pressure and central venous pressure monitoring are recommended. A transesophageal echocardiography is a very practical and instructive method for monitoring patients with volume problems or cardiac insufficiency. An arterial pressure wave contour based cardiac output and stroke volume variation monitoring is feasible; however, it is associated with decreased accuracy in patients with a very high heart rate or with a vasopressor drip.^[20]^ Special attention should be paid to the monitoring of neuromuscular function in patients with hyperparathyroid crisis. Papadima et al. reported a patient with hyperparathyroid crisis, found recovery of T1 to 25% of control of cisatracurium was decreased and response to neostigmine was rapid in TOF monitor.^[19]^ Al-Mohaya et al demonstrated the same results for atracurium in patients with hyperparathyroidism.^[21]^

Although the first chest X-ray after admission was clear, our patient developed aspiration pneumonia before surgery. Nausea, vomiting, and cognitive disturbance, which are risk factors of aspiration pneumonia, were noted in our patient. Yu et al reported that a semicomatosed patient experienced severe nausea and vomiting, which resulted in aspiration pneumonia, and eventually died of respiratory failure after surgery for hyperparathyroid crisis.^[9]^ In addition, Bentrem et al. showed that 18% of patients with hyperparathyroidism had thyroid disease^[22]^ and the percentage of cases of pancreatitis among patients with hyperparathyroid crisis was significantly greater.^[3,11]^ These concomitant diseases are often ignored and should be brought to the attention of the anesthesiologist during the preoperative evaluation.

Calcium activates several factors in the clotting system. This may, in part, account for occasional reports of thrombotic events (stroke, pulmonary embolism, or deep venous thrombosis) in patients with hyperparathyroidism.^[23]^ A thrombotic event also occurred in our patient. The hemodialysis catheter had been blocked before surgery and was unclogged with urokinase. Postoperative ultrasonography revealed thrombi around the hemodialysis catheter and thrombosis in the calf intermuscular vein. Whether routine prophylactic anticoagulation is effective for patients with hyperparathyroid crisis is currently inconclusive. Given the various manifestations of hyperparathyroid crisis, an individualized anticoagulant treatment plan might be better.

In conclusion, parathyroidism crisis is a disease involving multiple organs and systems, with diverse clinical manifestations. Anesthesiologists should pay attention to the timing of surgery, the airway, volume status, control of serum calcium level, organ function, and coagulation in anesthetic management during the perioperative period. Thyroid disease, pancreatitis, and aspiration pneumonia are common concomitant diseases, and if neglected, could result in severe and lethal outcomes.

## Author contributions

**Conceptualization**: Hao Kong, Hong Zhang

**Investigation**: Hao Kong, Zhen Zhang

**Supervision**: Hong Zhang

**Writing – original draft**: Hao Kong, Zhen Zhang

**Writing – review and editing**: Hao Kong, Hong Zhang
